# Effectiveness of a Balanced Nine-Strain Synbiotic in Primary-Care Irritable Bowel Syndrome Patients—A Multi-Center, Randomized, Double-Blind, Placebo-Controlled Trial

**DOI:** 10.3390/nu16101503

**Published:** 2024-05-16

**Authors:** Henning Sommermeyer, Krzysztof Chmielowiec, Malgorzata Bernatek, Pawel Olszewski, Jaroslaw Kopczynski, Jacek Piątek

**Affiliations:** 1 Department of Health Sciences, Calisia University, Nowy Swiat 4, 62-800 Kalisz, Poland; drpiatek@o2.pl (M.B.); pawel.r.olszewski@gmail.com (P.O.); jkopczynski106@gmail.com (J.K.); drpiatek@interia.eu (J.P.); 2 Department of Hygiene and Epidemiology, Collegium Medicum, University of Zielona Góra, 28 Zyty St., 65-046 Zielona Góra, Poland; k.chmielowiec@inz.uz.zgora.pl

**Keywords:** gut microbiota, probiotics, abdominal pain, diarrhea, constipation, bowel habit, ViIBS trial

## Abstract

The aim of the study was to characterize effects of a multi-strain synbiotic in patients with moderate to severe irritable bowel syndrome (IBS) of all stool form types. A total of 202 adult IBS patients were randomized (1:1) and after a four-week treatment-free run-in phase and were treated either with the synbiotic or a placebo for 12 weeks. The primary endpoints were the assessment of the severity of IBS symptoms (IBS-SSS) and the improvement of IBS global symptoms (IBS-GIS). Secondary endpoints comprised adequate relief (IBS-AR scale), stool form type (Bristol Stool Form Scale), bowel movements, severity of abdominal pain and bloating, stool pressure, feeling of incomplete stool evacuation, and adverse events. A total of 201 patients completed the study. Synbiotic treatment, in comparison to placebo, significantly improved IBS-SSS and IBS-GIS scores. At the end of the treatment, 70% of patients in the synbiotic group achieved adequate relief. After 12 weeks of treatment, the secondary endpoints were favorably differentiated in the synbiotic group when compared with the placebo group. Two patients in the synbiotic group reported transient adverse events (headache). The results indicate that treatment of IBS patients with the synbiotic significantly improved all major symptoms of IBS and was well-tolerated. The ClinicalTrials.gov registration was NCT05731232.

## 1. Introduction

Irritable bowel syndrome (IBS) is a common and chronic functional gastrointestinal disorder (FGID), characterized by recurrent abdominal pain, bloating, and changes in stool form and frequency [1]. The condition is non-fatal, but the troublesome and unpredictable character of IBS symptoms has a negative impact on patients’ quality of life and work productivity [2,3]. The consistent health concerns of IBS patients can lead to anxiety and depression [4]. The disease results in increased healthcare utilization and healthcare costs [5,6]. Diagnosis of IBS is based solely on patients’ reported symptoms, as no reliable biological marker of IBS has yet been identified [7,8,9], and by the predominant stool pattern assessed by using the Bristol Stool Form Scale: IBS with diarrhea (IBS-D), IBS with constipation (IBS-C), mixed IBS (IBS-M), or untyped IBS (IBS-U) [10]. For the latter there is insufficient abnormality of stool consistency to meet criteria for IBS-C, D, or M. The pooled prevalence of IBS in the general population is about 11.2% [11]; however, it varies substantially among regions and the diagnostic criteria applied [2]. IBS manifests during adolescence, with females being more frequently affected than males [12].

The etiology of IBS is not fully understood, but the condition is assumed to be multifactorial, with environmental, inherited, and psychosocial factors contributing to disease manifestation. Suggested mechanisms include visceral hypersensitivity; dysfunction of the gut–brain axis; disturbances of the integrity of the gut epithelial barrier, leading to an abnormal intestinal permeability; altered gastrointestinal motility; activation of the immune system; disturbance of the enteroendocrine signaling; and dysbiosis of the gut microbiota [13,14]. Alteration of the gut microbiome as a potential cause or contributor to IBS has attracted increased attention, as the bacteria composition of IBS patients differs from those of healthy controls [15,16,17]. Specific gut-microbiota profiles have been associated with particular symptoms and the severity of diseases [18,19]. Recent years have seen a steady increase in the number of publications reporting results from clinical trials on IBS patients investigating the effects of probiotics, prebiotics, and synbiotics [20].

As the gut microbiota seems to play a crucial role in IBS, altering the composition of the gut microbiota of IBS patients by supplementation with products containing probiotic bacteria is considered a potential strategy for IBS management. Numerous single-strain and multi-strain probiotics or synbiotics (products containing probiotic bacteria and a prebiotic component) have been evaluated in randomized clinical trials. As with conventional drug interventions, the treatment of IBS patients with probiotics has been shown to improve at least some of the IBS symptoms [21]. A number of recent meta-analyses and systematic reviews have evaluated the effects of products containing probiotic bacteria in IBS patients [20,22,23,24,25,26,27,28]. However, determining the therapeutic value of these products is inherently complex, due to the large diversity of studied products (mono-, multi-strain products, probiotics, and synbiotics), the variety of study designs, and the often small number of patients investigated in the trials. While the results of these studies should be interpreted with care, at least some mono-strain and multi-strain products seem to have beneficial symptom-improving effects in IBS patients. The results also revealed that the benefits provided by probiotics are strain specific rather than species specific. While some analyses have found that multi-strain products are more effective than mono-strain products [24,25], others found no superiority [27,28]. So far, the relatively limited number of clinical trials investigating synbiotic therapies in IBS patients have produced varying results. This could be due to the different mixtures of probiotic and prebiotic components that have been investigated in the trials, as well as the different subsets of IBS patients enrolled in these studies [23,29,30,31]

The multi-strain synbiotic mixture evaluated in the present clinical trial contains a mixture of nine well-characterized probiotic bacteria strains in an enteric-coated capsule. The composition of the probiotic bacteria mixture is balanced, as each of the individual strains contributes between 5 to 20%, without any strain dominating the mixture as is sometimes found in multi-strain products. The protection of the probiotic bacteria against inactivation by the low pH of the stomach by the special enteric-coated capsule has been demonstrated in in vitro experiments [32]. The bacteria of the mixture adhere strongly to intestinal epithelial cells as has been illustrated by using the Caco-2 cell adhesion model [32]. Inhibition of pathogenic bacteria by the mixture has been shown for *Salmonella typhimurium*, *Klebsiella pneumoniae*, and *Clostridioides difficile* [33,34,35]. Since its market introduction as a food supplement in Germany in 2013, more than 13.5 million daily doses have been dispensed. During the last ten years, numerous anecdotal reports have been collected from patients, pharmacists, and physicians, suggesting that the product might have beneficial effects on IBS patients. Triggered by these anecdotal reports, the present study aimed to characterize the effects of this multi-strain synbiotic in IBS patients under the rigorous conditions of a multicentric, randomized, placebo-controlled clinical trial in primary-care IBS patients with moderate to severe disease, independent of their stool characteristics.

## 2. Materials and Methods

### 2.1. Study Design

**Figure 1:**
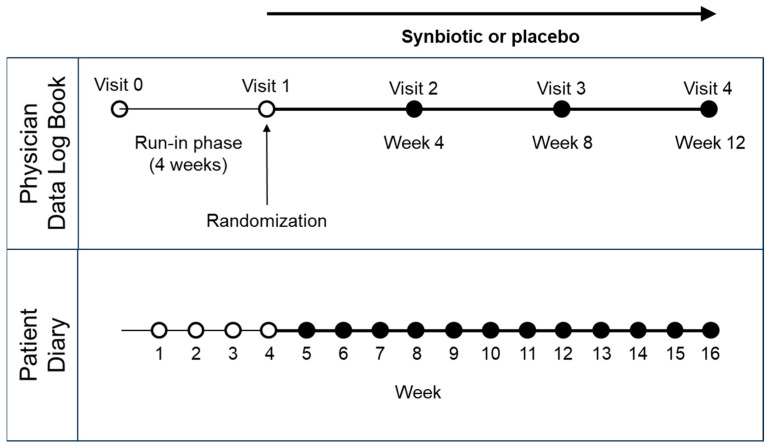
Study design. Data from physicians’ examinations were collected in a data logbook during the enrollment visit (V0) and following visits (V1 to V4) every four weeks. Patients reported symptoms on a weekly basis in a patient diary.

The ViIBS (Vivatlac in irritable bowel syndrome) study design was a multi-center, randomized, double-blind, placebo-controlled, two equal size parallel arms clinical trial. The study design was inspired by those of two recently published IBS studies [36,37]. The trial design is summarized in Figure 1.

Patients were enrolled at the family doctor’s clinic, in 62-820 Stawiszyn, West Poland and at the family doctor’s outpatient clinic “Panacea”, in 27-230 Krynki, East Poland by a total of eight physicians. The study was advertised to patients visiting the study centers. Interested patients were informed about the details by their physician. Patients were informed that they had a 50% chance to be allocated to the placebo arm of the trial and that the verum might not have any beneficial effects in regard to their IBS condition. Each individual patient was cared for from the start to the end of the trial by one physician only. The first patient was enrolled in February 2023, and the treatment of the last patient finished in November 2023. Patients willing to participate were asked to sign an informed consent. Those who signed the informed consent were enrolled in the study by the physician. Neither patients nor physicians were incentivized to participate in the trial. The study protocol was approved by the Ethics Committee of Calisia University (project identification code 1/2023). The trial was conducted in accordance with the Declaration of Helsinki and was registered at ClinicalTrials.gov (NCT05731232).

### 2.2. Study Participants

The study recruited female and male patients aged 18 to 65 years diagnosed with irritable bowel syndrome according to the IBS questionnaire for healthcare providers (HCP) developed by the World Gastroenterology Organization (WGO) [38] and having an IBS severity, assessed by using the IBS-SSS, of ≥175 points, representing moderate to severe cases of IBS [8].

The exclusion criteria included the use of products containing probiotic bacteria or treatment with antibiotics within the last three months; concurrent severe illness (malignancies, uncontrolled hypertension or diabetes, hepatic, renal, or cardiac dysfunctions, serious neurological disorders, psychosis, respiratory disorders such as asthma or chronic obstructive pulmonary disease, and hyper- or hypothyroidism), chronic bowel disorders other than IBS, including inflammatory bowel disease, gastroenteritis, stomach and duodenal cancer, celiac disease, pregnancy or lactation; diagnosed lactose intolerance; use of motility drugs or dietary fiber supplements within two weeks before the study start; taking anti-coagulant medication; and participation in another clinical trial within the last three months. Patients who received antibiotics during the study were excluded.

### 2.3. Multi-Strain Synbiotic and Placebo Preparation

The synbiotic preparation investigated in the present study is a mixture of nine probiotic bacterial strains and fructooligosaccharides (FOS) as a prebiotic component (Table 1).

Each individual probiotic strain of the mixture contributes between 5 to 20% of the total colony-forming units (4.5 × 10^9^ per capsule), representing a balanced mixture without the dominance of one probiotic bacteria strain. The preparation is commercially available (Vivatlac^®^ Synbiotikum, Vivatrex^®^ GmbH, Groiner Kirchweg 68, 46459 Rees, Germany) and was purchased for the trial. The probiotic bacteria of the product are protected against inactivation by the low pH of the stomach by a special enteric coating of the capsule [32]. The placebo capsules were identical in appearance, size, and weight and contained maize starch produced by Solger SP (Kamienica 40, 62-530 Kamienica, Poland). To ensure the blinding of patients and physicians, the placebo and the multi-strain synbiotic were blinded by packaging in white plastic bottles individually labeled with sequential trial codes according to the randomization list created for the two treatment arms of the trial. Neither physicians, assisting nurses, nor patients were aware of the treatment arms and drug codes. Each bottle contained 84 capsules covering the 12-week treatment period for each trial participant. Unblinding of the data was performed only after the data of all patients had been transferred into the study database.

### 2.4. Examinations, Treatments, and Data Collection

During an initial visit (visit 0), the patients underwent an in-depth physical examination, diagnosis for IBS using the World Gastroenterology questionnaire for HCP [38], determination of IBS severity using the IBS-SSS [8], determination of the type of IBS using the Bristol Stool Form Scale [10], and a check of inclusion and exclusion criteria. Physicians reported all results of their examinations in a printed physicians’ data logbook. During visit 0, the patients were provided with a patient’s diary and instructed on how to use it for symptom reporting on a weekly basis.

At the end of a four-week run-in phase without treatment, the assessment of the severity of IBS symptoms using the IBS-SSS was repeated (visit 1). Global improvement was evaluated by using the IBS Global Improvement Scale (IBS-GIS) [39], and assessment of adequate relief was performed with the IBS-Adequate Relief Scale (IBS-AR) [40]. During visit 1 the quality of the patients’ reporting (e.g., all questions answered?) was checked by the physician, and in case it was not satisfactory, patients were retrained on how to use the diary. At the end of visit 1, the physician assigned treatment according to the randomization list to the patient based on the patient’s trial entrance number.

The treatment duration was 12 weeks, with the administration of one capsule per day. During the treatment phase, the patients were examined every four weeks (visits 2 to 4), assessing the severity of symptoms (IBS-SSS), global improvement (IBS-GIS), and adequate relief (IBS-AR scale). In addition, the physicians checked for the occurrence of adverse events. The examination results were protocolled by physicians in the data logbooks. During each of the visits, physicians checked the patient diary for completeness and reporting of adverse events. The patient diaries were collected from the patients by the physicians during the visit at the end of the treatment period (visit 4).

After the patients had finished their trial participation, the physician’s data logbooks and patients’ diaries were shipped to the trial manager in charge of transferring the data to a database. Data were unblinded only after the data from all patients who completed trial participation had been added to the database.

### 2.5. Sample-Size Calculation

The necessary sample size was calculated assuming a significance level of 5%, a power of 90%, a responder rate of 25% in the placebo group and 50% in the multi-strain synbiotic group, and a dropout rate of 25%. Using the Sample Size Calculator of Sealed Envelope Ltd. (London, UK), a required sample size per treatment arm of 100 patients was determined.

### 2.6. Randomization

A two-treatment equal allocation (1:1) randomization scheme using a block size of four was generated by the trial statistician using the Simple Randomiser of Sealed Envelope Ltd. (London, UK). Treatment was allocated to patients by physicians using the randomized scheme based on the patient’s entry position into the study.

### 2.7. IBS-Type Categorization

Patients were categorized as IBS-D patients when they reported only Bristol Stool Form Scale scores of five, six, or seven [10] during the four-week run-in phase. If patients reported only scores of one or two, they were categorized as IBS-C patients. Patients reporting at least one five, six, or seven score and at least a single one or two score during the screening phase were categorized as IBS-M types. Patients who reported at least one score of three or four during the first four weeks or patients without the full set of four scores were categorized as IBS-U patients.

### 2.8. Primary Endpoints

Primary efficacy endpoints defined for the trial were (i) assessment of the severity of IBS symptoms using the IBS-SSS and (ii) assessment of improvement or worsening of IBS global symptoms using the IBS-GIS.

IBS-SSS is a five-question survey evaluating for the last 10 days (1) the severity of abdominal pain (IBS-SSS1), (2) the frequency of abdominal pain (IBS-SSS2), (3) the severity of abdominal flatulence (IBS-SSS3), (4) the dissatisfaction with bowel habit (IBS-SSS4), and (5) the interference with quality of life (IBS-SSS5) [8]. Patients responded to each question on a 100-point visual analog scale. Each of the five questions generated a maximum score of 100 points, and the total scores could range from 0–500, with higher scores indicating severe symptoms or a higher burden for patients. When registering the trial, we assumed, as done by many others, that patients with decreases of ≥50 points on the IBS-SSS at visit 4 compared to the value at visit 0 could be considered to be responders [8].

With the IBS-GIS, global improvement is assessed by using a patient-defined 7-point Likert scale ranging from symptoms substantially worse (1 point) to substantially improved (7 points) [39]. Patients answered the question “Have you felt any change in the severity of your symptoms over the past 7 days compared to how you felt before the medication was taken?” Answers were recorded based on the 7-point scale: 1 point—“I feel that the symptoms have worsened significantly”; 2 points—“I feel that the symptoms have moderately worsened”; 3 points—“I feel that the symptoms have slightly worsened”; 4 points—“I feel no change”; 5 points—“I feel a slight improvement”; 6 points—“I feel a moderate improvement”; 7 points—“I feel a significant improvement”. The IBS-GIS score indicated an improvement if the score was ˃4, worsened if it was ˂4, and no change if it was 4. Patients for which a score increase on the IBS-GIS of 3 points was reported were considered as responders. For information purposes, the percentage of patients with an improvement in the IBS-GIS score of 2 or more points was also calculated.

### 2.9. Secondary Endpoints

Secondary efficacy endpoints included the IBS-Adequate Relief (IBS-AR), which is a dichotomous single item that asked trial participants “Over the past week have you had adequate relief of your IBS-symptoms?” [40]. The answer was either “yes” or “no”. IBS-AR scores were assessed during visits 1 to 4.

Other secondary efficacy endpoints were (i) the type of stool assessed using the Bristol Stool Form Scale [10], (ii) the frequency of bowel movements per day, (iii) the severity of pain, (iv) the severity of flatulence, (v) stool pressure, (vi) the feeling of incomplete evacuation of stool, and (vii) adverse events. All secondary endpoint information was collected by the use of a patient diary that patients used to report on a weekly basis during the 4 weeks of the run-in phase and the 12 weeks of the treatment phase.

The stool types were assessed by using the Bristol Stool Form scale (visualized in the patient diary), which classifies feces into seven groups: types 1–2 indicate constipation; types 3–4 are normal stools, and types 5–7 indicate diarrhea.

IBS symptoms, except for a feeling of incomplete bowel movement, were assessed using a patient-defined 5-point Likert scale, with 0 points indicating no symptoms and 1–4 indicating the severity of symptoms, and higher scores indicate worse symptoms. The feeling of incomplete bowel movement was assessed using a 2-point scale: 0—no such feeling and 1—there is an incomplete bowel movement. Adverse events were evaluated on a two-item scale (yes or no) and a field for free text to describe the nature of any adverse event.

### 2.10. Statistical Analyses

For quantitative variables, basic statistics, such as mean and standard deviation, were calculated. Percentages are given for the frequency variables.

Changes in the severity of IBS symptoms assessed using the IBS Severity of Symptoms Scale (IBS-SSS) in the weeks between V0 and V4 were compared between the placebo group and the multi-strain synbiotic group using the Student’s *t*-test. This test was used to compare weight (kg), height (cm), body-mass index (kg/m^2^), age, and score on the WGO IBS questionnaire for HCPc. Similarly, when comparing the type of stool in patients with IBS-D and IBS-C assessed using the Bristol Stool Form Scale, the average number of bowel movements per day, severity of abdominal pain, severity of bloating, and stool pressure reported by patients on a 5-point Likert scale, Student’s *t*-test was used between the placebo group and the multi-strain synbiotic group. The calculated power for the Student’s *t*-test was 0.97 (alpha = 0.05, n1 = 100, n2 = 101, IBS-SSS: M1 = 295.1, M2 = 300.5, and critical value t = 1.972).

The differences between the value determined in the first week and the value measured in later weeks (from 1 to 16) separately for the placebo group and the multi-strain synbiotic group were calculated using a one-way variance for the following characteristics: change of stool form type in patients with IBS-D and IBS-C assessed with the Bristol Stool Form Scale, average number of bowel movements per day, severity of abdominal pain, and severity of bloating and stool pressure reported by patients on a 5-point Likert scale. Also, a one-way analysis of variance was used to analyze the differences in the IBS SSS and the IBS-GIS scores determined for the placebo and the multi-strain synbiotic groups during the physicians’ examinations (V0 to V4). The condition of homogeneity of variances was met (Levene’s test *p* > 0.05). A post hoc Fisher’s last significant differences (LSD) test was performed for multiple comparisons. The calculated power for the ANOVA test was 0.96 (alpha = 0.05, number of groups = 4, sample size n = 100, root mean square standardized effect = 0.25, degrees of freedom of effect = 3, degrees of freedom of error = 396, and critical value F = 2.627).

To demonstrate differences in frequency variables, such as gender (female/male), IBS severity (moderate/severe), percentage of patients with adequate relief assessed with the IBS-AR, and percentage of patients reporting a feeling of complete evacuation of stool between the placebo group and the multi-strain synbiotic group, the Chi2 test was used.

The above calculations were performed using STATISTICA 13 (version 13.3.x, Tibco Software Inc., Palo Alto, CA, USA) for Windows (Microsoft Corporation, Redmond, WA, USA). For all statistical tests used, the level of statistical significance was *p* ≤ 0.05.

## 3. Results

### 3.1. Patient Flow, Study Progress, and Baseline Characteristics of Treatment Groups

**Figure 2:**
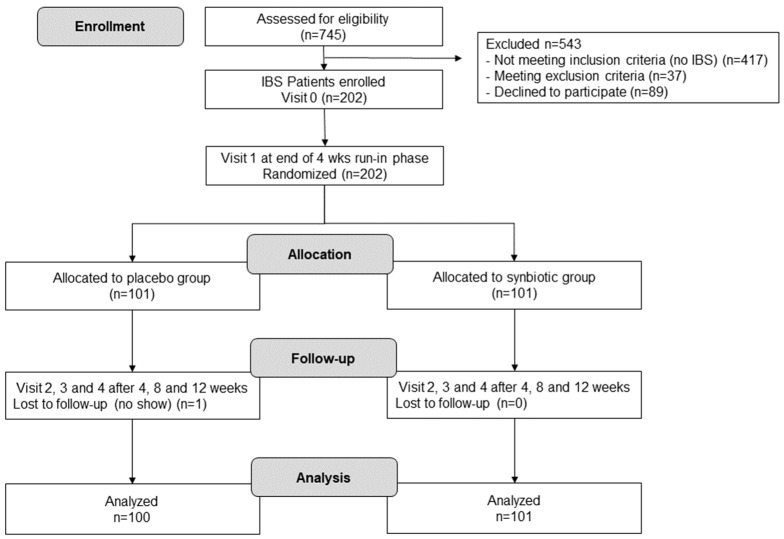
Patient enrollment and study progress.

Figure 2 shows the flow of the patients through the study. A total of 745 patients were assessed for eligibility. Of these, 328 were diagnosed as IBS patients using the WGO questionnaire for HCP. A total of 37 IBS patients had to be excluded from the study, as they met at least one of the exclusion criteria. Of the remaining 291 IBS patients, 89 declined to participate. The main reasons for not being interested in participating were that no incentives for participating were provided, the chance to receive a placebo was 50%, and the information that the verum might not result in improvements in the IBS condition of the patient. For the run-in phase, 202 patients were enrolled. No patient was lost during the run-in phase and all of the 202 patients were randomly allocated (1:1) to the placebo or the multi-strain synbiotic group. One patient was lost during the treatment phase (no show). Unblinding later revealed that the lost patient belonged to the placebo group. Data from 100 patients of the placebo arm and 101 patients of the multi-strain synbiotic arm were analyzed.

The baseline characteristics of patients of the placebo group and the multi-strain synbiotic group are shown in Table 2. The ratio between female and male study participants was 1.45. Patients with moderate IBS at the beginning of the study represented 54.7% of all participants. IBS with diarrhea was diagnosed in 72.6% and IBS with constipation in 22.9% of patients. Statistical analyses revealed that there were no significant differences between the two groups with respect to weight, height, body-mass index, gender, the share of IBS stool subtypes, and IBS severity (total IBS-SSS score). The statistically significant difference between the two groups (*p* < 0.05) was identified for the scores of the IBS-SSS subscale 3 (severity of flatulence) and IBS-SSS subscale 5 (interference with quality of life), which were in both cases higher in the multi-strain synbiotic group.

### 3.2. Study Primary Endpoints

**Figure 3:**
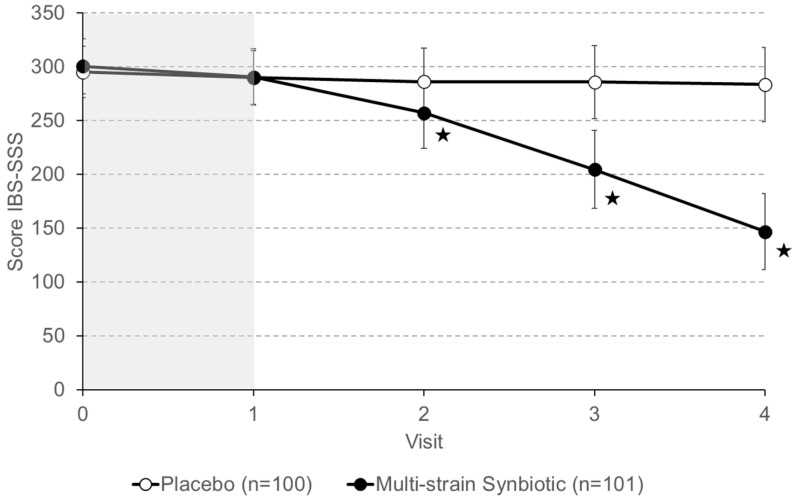
Change of IBS symptom severity assessed with the IBS Severity of Symptoms Scale (IBS-SSS). Data shown are means ± S.D. The 4-week treatment-free run-in phase (visit 0 to 1) is highlighted in grey. Examinations by physicians were performed every 4 weeks. Asterisks (★) indicate statistically significant differences (*p* < 0.05) between the placebo group and the multistrain synbiotic group.

Changes in IBS-symptoms severity using the IBS-SSS were one of the two primary endpoints defined for the trial (Figure 3). The total IBS-SSS scores at the beginning of the run-in phase (visit 0) were similar in both groups: 295.1 ± 23.9 (mean ± standard deviation) and 300.5 ± 25.7 in the placebo and multi-strain synbiotic groups, respectively. At the end of the run-in phase, these values had slightly decreased to 289.8 ± 25.0 and 290.4 ± 26.2 in the placebo and multi-strain synbiotic groups, respectively. Treatment with the multi-strain synbiotic resulted in a progressing reduction of IBS symptom severity, while such an effect was not observed in patients receiving a placebo.

Using a reduction of ≥50 of the IBS-SSS score as the cutoff for the calculation of an IBS-SSS responder rate at the end of the 12-week treatment period (visit 4) versus the baseline value (visit 0) resulted in a 14% responder rate for the placebo group and 98% for the multi-strain synbiotic group.

**Figure 4:**
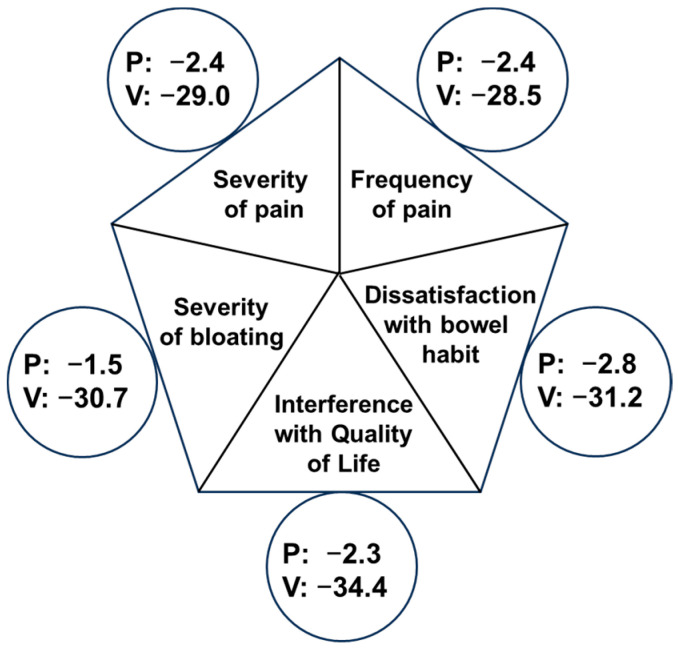
Absolute changes of IBS Severity of Symptoms subscales (IBS-SSS1 to 5) measures at the end of treatment (visit 4). Shown are absolute changes (mean score at V4 minus mean score at V0) of the individual subscale scores of the IBS-SSS for the group of patients treated with placebo (P) or the multi-strain synbiotic (V). At visit 4, all mean subscale scores determined for the multi-strain synbiotic group were significantly different (*p* ˂ 0.05) from those of the placebo group.

Analyses of the IB-SSS domain-specific scores IBS-SSS1 to IBS-SSS5 (secondary endpoints) showed that the multi-strain synbiotic caused decreases in the scores of all sub-scales. An overview of the effects on the individual IBS-SSS subscales at the end of the treatment (visit 4) is shown in Figure 4. As is visible, the multi-strain synbiotic improves all five subscale measures of the IBS-SSS to a similar extent. Time progressions of the score reductions were similar for all individual subscales.

**Figure 5:**
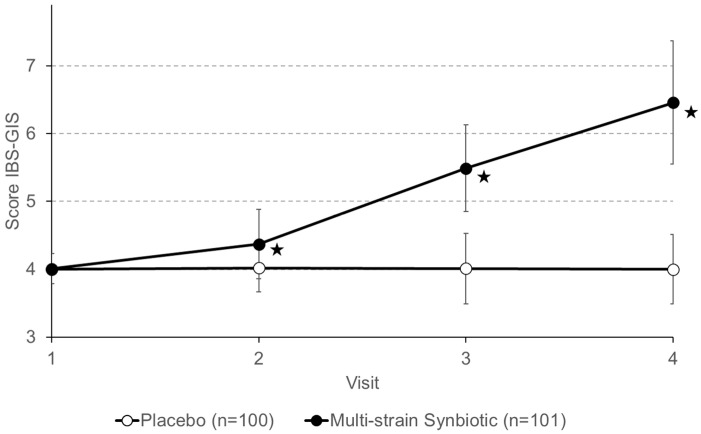
Global improvement of IBS determined with the IBS Global Improvement Scale (IBS-GIS). Data shown are means ± S.D. Scores above 4 indicate improvement, below 4 indicate worsening, and 4 indicates no change. Examinations by physicians were performed every 4 weeks. Asterisks (★) indicate statistically significant differences (*p* < 0.05) between the placebo group and the multistrain synbiotic group.

Global improvement assessed with the IBS-GIS was the second primary endpoint of the trial (Figure 5). At the end of the run-in phase (visit 1), no change in the IBS-GIS was reported for either patients from the placebo group or patients from the multi-strain synbiotic group. There were no changes in IBS-GIS scores caused by treatment with a placebo (visit 2 to 4). For the multi-strain synbiotic group, the IBS-GIS score steadily increased with the progression of treatment duration (visits 2 to 4). At the end of the treatment period (visit 4), 52.5% of patients of the multi-strain synbiotic group experienced a change in the IBS-GIS of three points and 89.1% a change of at least two points.

### 3.3. Study Secondary Endpoints

**Figure 6:**
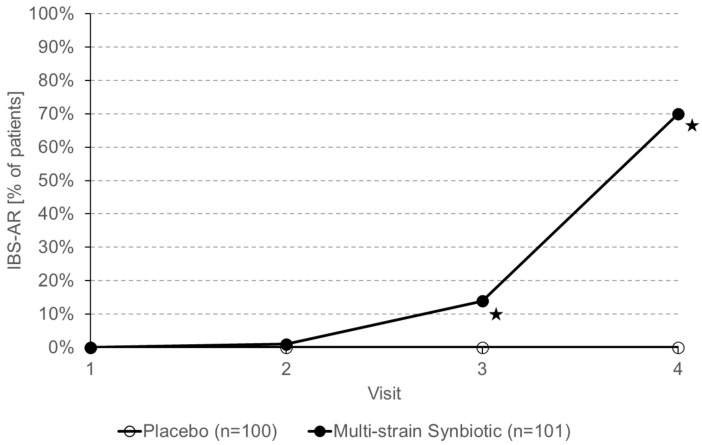
Percentage of patients with adequate relief assessed with the IBS-AR. Asterisks indicate statistically significant differences (*p* < 0.05) between the placebo group and the multi-strain synbiotic group.

Adequate relief assessed with the IBS-AR Scale was defined as one of the secondary trial endpoints. As shown in Figure 6, no patient in the placebo group experienced adequate relief. In the multi-strain synbiotic group, 70% of patients achieved adequate relief after 12 weeks of treatment. After 4 and 8 weeks of treatment with the multi-strain synbiotic, adequate relief was achieved by 1% and 14% of patients, respectively.

**Figure 7:**
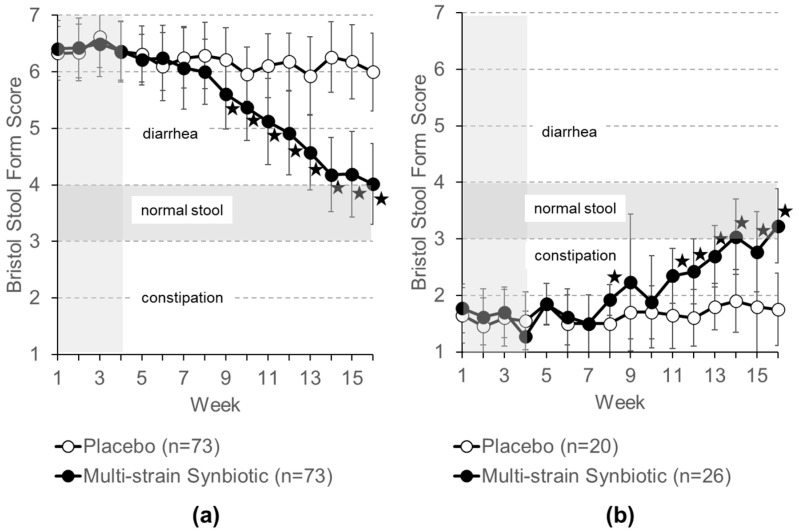
Weekly patient self-reported Bristol Stool Form scores of (**a**) IBS-D patients and (**b**) IBS-C patients. Data shown are means ± S.D. The 4-week treatment-free run-in phase (weeks 1 to 4) is highlighted in grey. Bristol Stool Form scores of 5–7 indicate diarrhea, 1–2 constipation, and 3–4 normal stool forms. Asterisks (★) indicate statistically significant differences (*p* < 0.05) between the placebo group and the multistrain synbiotic group.

Results from the self-assessment of stool consistency by patients using the Bristol Stool Form Scale are shown in Figure 7a for IBS-D patients and in Figure 7b for IBS-C patients. At the end of the treatment period, 78.1% of the IBS-D patients (n = 73) receiving the multi-strain synbiotic and 2.7% of those receiving placebo (n = 73) reported a Bristol Stool Form score of four or lower. Of the IBS-C patients, 96.2% of those treated with the multi-strain synbiotic (n = 26) and 10.0% of those treated with the placebo (n = 20) reported a score of three or higher.

During the four-week run-in phase, the patient self-reported number of average bowel movements per day was 3.2 ± 0.7 (mean ± S.D.) in the multi-strain synbiotic and 3.3 ± 0.8 (mean ± S.D.) in the placebo group, respectively. During the last four weeks of the treatment phase, these numbers were reduced to 1.5 ± 0.5 (mean ± S.D.) for the synbiotic treatment group and to 2.7 ± 0.7 for the placebo group (mean ± S.D.), respectively.

**Figure 8:**
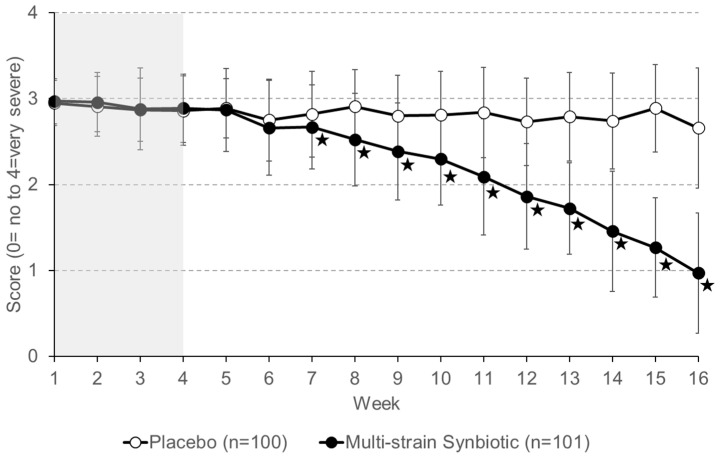
Severity of abdominal pain was self-reported by patients on a 5-point Likert scale. Data shown are means ± S.D. The four-week run-in phase (no treatment) is highlighted in grey. Asterisks indicate statistically significant differences (*p* < 0.05) between the placebo group and the multi-strain synbiotic group.

Patient self-reported abdominal pain severity for the placebo group and the multi-strain synbiotic group during the 4 weeks (weeks 1 to 4) of the run-in phase (no treatment) were similar (Figure 8). Treatment with a placebo resulted in no major change in the pain severity level compared to that reported for the run-in phase. In contrast, patients receiving the multi-strain synbiotic reported a gradual decrease in abdominal pain severity. At the end of the treatment phase (week 16), a greater than 50% reduction in abdominal pain severity compared to that reported during the run-in phase of the study (average of week 1–week 4) was stated by 1% of patients of the placebo group and 78.2% of the multi-strain synbiotic group, respectively.

**Figure 9:**
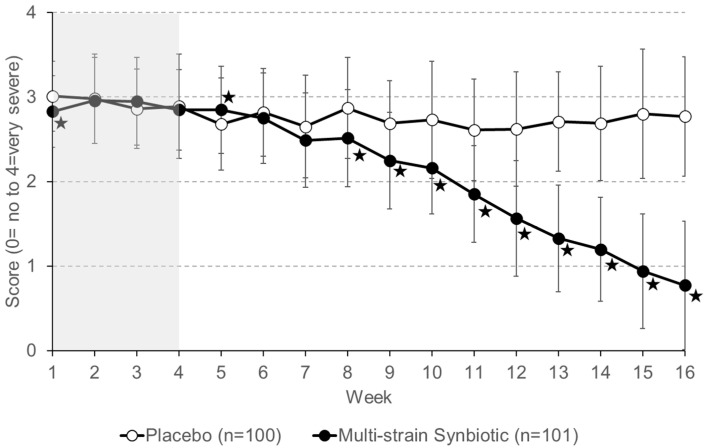
Severity of abdominal bloating self-reported by patients on a 5-point Likert scale. Data shown are means ± S.D. The four-week run-in phase (no treatment) is highlighted in grey. Asterisks indicate statistically significant (*p* < 0.05) differences between the placebo group and the multi-strain synbiotic group.

Patient self-reported severity of bloating of the placebo group and the multi-strain synbiotic group during the 4 weeks (weeks 1 to 4) of the run-in phase (no treatment) was similar (Figure 9). Treatment with a placebo resulted in no major change in the bloating severity level compared to that reported for the run-in phase. In contrast, patients receiving the multi-strain synbiotic reported a gradual decrease in bloating severity. At the end of the treatment phase (week 16), a greater than 50% reduction in bloating severity compared to that reported during the run-in phase of the study (average of weeks 1 to 4) was stated by 1% of patients of the placebo group and 81.2% of the multi-strain synbiotic group, respectively.

**Figure 10:**
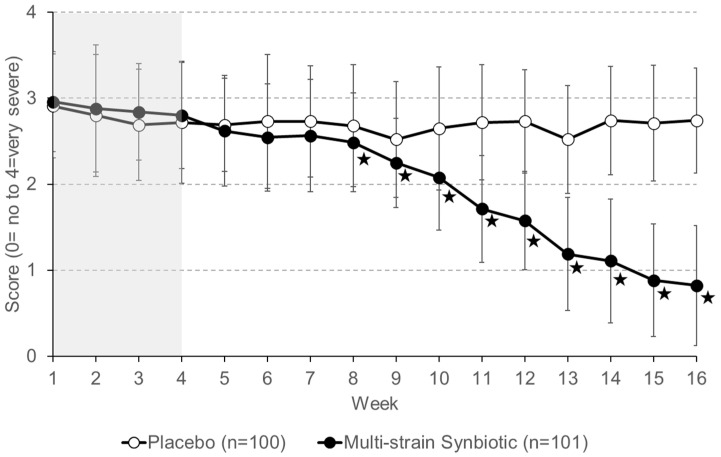
Stool pressure self-reported by patients on a 5-point Likert scale. Data shown are means ± S.D. The four-week run-in phase (no treatment) is highlighted in grey. Asterisks indicate statistically significant differences (*p* < 0.05) between the placebo group and the multi-strain synbiotic group.

Patient self-reported stool pressure of the placebo group and the multi-strain synbiotic group during the 4 weeks (weeks 1 to 4) of the run-in phase (no treatment) were similar (Figure 10). Treatment with a placebo resulted in no major change in stool pressure level compared to that reported for the run-in phase. In contrast, patients receiving the multi-strain synbiotic reported a gradual decrease in stool pressure. At the end of the treatment phase (week 16), a greater than 50% reduction of stool pressure severity compared to that reported during the run-in phase of the study (average of weeks 1 to 4) was stated by 1% of patients of the placebo group and 82.2% of the multi-strain synbiotic group, respectively.

**Figure 11:**
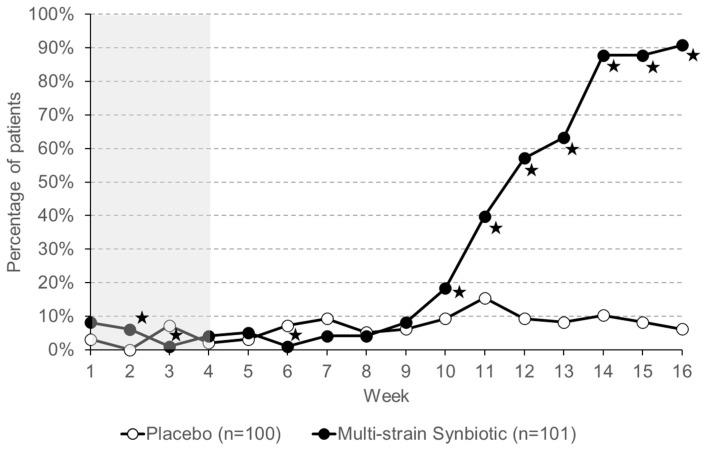
Percentage of patients having a feeling of complete evacuation of stool. Asterisks indicate statistically significant differences between the placebo group and the multi-strain synbiotic group. The four-week run-in phase (no treatment) is highlighted in grey. Asterisks indicate statistically significant differences between the placebo group and the multi-strain synbiotic group.

As shown in Figure 11, during the run-in phase of the trial, a feeling of complete evacuation of stool was reported by less than 10% of patients in the two treatment groups. While this percentage remains relatively stable in the placebo patient group, there was a gradual increase in patients from the multi-strain synbiotic group reporting a feeling of complete stool evacuation. This increase started to become visible only after six weeks of taking the multi-strain synbiotic. At the end of the treatment phase, 90% of the patients who had received the multi-strain synbiotic reported a feeling of complete stool evacuation.

None of the patients treated with placebo reported adverse events. Two patients in the multi-strain synbiotic group reported mild headaches. Both patients experienced mild headaches for two non-consecutive weeks in the last third of the treatment phase. Despite the mild headache, both patients completed their trial participation.

## 4. Discussion

Compared to treatment with placebo, the multi-strain synbiotic exhibited significant beneficial effects in terms of IBS-symptom severity (IBS-SSS score reduction) and the global improvement of symptoms (IBS-GIS score increase). Both measures had been defined before the start of the study as primary endpoints. Patients receiving the multi-strain treatment also experienced superior improvements in the measures defined as secondary trial endpoints. While after 12 weeks the effects obtained by treatment with the synbiotic were significantly superior to those observed in the placebo group, it has to be noted that the effects needed some time to manifest and were hardly visible during the first four weeks of the treatment phase of the trial. The results of this clinical trial confirm earlier anecdotal reports from patients, pharmacists, and physicians about the beneficial effects of this particular synbiotic preparation in IBS patients. An interesting and new insight resulting from the study is the fact that the tested multi-strain synbiotic exhibited its effects more or less equally across all major symptoms of IBS.

The effects of probiotic preparations observed in randomized clinical trials with IBS patients have been summarized in a number of systematic reviews and meta-analyses published recently [20,26,27,31]. However, the interpretation of these systematic reviews and meta-analyses is limited for a number of reasons. First, the probiotic preparations evaluated in the analyzed trials vary widely in regard to the types of probiotic strains, numbers of strains, amounts of CFUs administered, and protections of the strains against inactivation by stomach acid. Second, there is little standardization of trial design (e.g., used trial endpoints), some of which are, additionally, of limited quality. Third, the number of patients involved in the trials rarely exceeds 100. Fourth, the treatment duration of some trials is so short (less than 4 weeks) that this on its own might influence the observed clinical trial outcomes.

There is a limited number of randomized clinical trials that have evaluated the effects of synbiotics in IBS patients [36,41,42,43,44,45,46]. Summarizing the results of these studies is difficult, as study designs and study endpoints vary significantly. However, in none of these studies did the observed improvements cover a broader range of the main IBS symptoms. Either improvement in pain [42,43] or improvement in flatulence/bloating [36,45] was observed. The only study failing to show any beneficial effects of a synbiotic administration suffered from a short treatment duration (2 weeks), which might be the reason for the negative outcome of the trial [44]. Consequently, reviews of these trials came to the conclusion that the results obtained with synbiotics are promising but that the currently available data are sparse and do not allow for a solid assessment of the role of synbiotics in the treatment of IBS [29,31]. Whether synbiotics are superior to probiotics remains unclear.

Of particular interest for the discussion of the results obtained in the present study are the trial results published by Skrzydlo-Radomska et al., as their study design was used as a reference when the study protocol of the present trial was developed [36]. While the same endpoints were used, the present study employed a higher number of IBS patients (201 versus 68), included IBS patients of all stool types (in contrast to the restriction to IBS-D patients), and observed treatment effects over a longer time period (12 weeks versus 8 weeks).

As done by Skrzydlo-Radomska et al., the present study started with a run-in phase (four weeks with no treatment), allowing for the establishment of baseline values for all measures collected during the trial. During the run-in phase, hardly any changes in the measures employed by the study were observed, indicating that enrollment into the study by itself had little impact on the patients. Administration of the synbiotic resulted in significant improvements in the trial’s primary endpoints (IBS-SSS and IBS-GIS) when compared with the baseline values and with those of the placebo group. However, the effects of the treatment with the synbiotic developed gradually over time. First, moderate improvements became visible after 4 weeks, with significantly better effects in the synbiotic treatment group compared to the placebo group observed after 8 weeks. Further increases in effect sizes in the synbiotic treatment group were observed after 12 weeks of treatment. A potential explanation for the gradual onset of effects might be that the administered synbiotic influences the gut microbiota and that this is a time-demanding process. However, as no analyses of the fecal microbiota were part of the present study, this interpretation remains purely speculative. In contrast to numerous other clinical trials with IBS patients, the placebo effects observed in the current study were moderate. A potential explanation for this observation could be the fact that the trial was performed as part of the normal care provided to patients in the primary-care setting of the study centers. Contact with patients by healthcare professionals during the trial was limited to the five physician visits, which were hardly longer than normal physician visits executed in the centers. Frequent contacts with patients and intensive caretaking during clinical IBS trials have been considered as factors that might result in high placebo effects, as they might provide patients with a form of psychological support, resulting in positive effects even in patients treated with a placebo [36,47].

The overall reduction in the IBS-SSS observed for the synbiotic treatment group in the present study was caused by significant reductions in all five IBS-SSS subscale measures (IBS-SSS1 to IBS-SSS5). In contrast to other IBS trials characterizing synbiotics in adult IBS patients, the multi-strain synbiotic evaluated in the present trial exhibits significant beneficial effects in all symptomatic areas of importance for IBS patients (abdominal pain, flatulence, dissatisfaction with bowel habit, and interference with quality of life). In addition, it normalizes the stool pattern in IBS-D and IBS-C patients. The reason for this broad range of effects in IBS patients remains unclear, as no information about the mechanism of action of the individual probiotic strains or potential synergistic effects among them is available. Some recent reviews analyzing the effects of probiotics on IBS patients suggested that multi-strain probiotic mixtures might have a more robust effect on improving IBS symptoms than mono-strain probiotics [21,24,25,26,27,28,48]. However, so far, the evidence is too limited to prefer multi-strain probiotics or synbiotics over mono-strain products for IBS treatment.

The present study saw two patients of the multi-strain synbiotic group experiencing transient episodes of headaches as adverse events. Despite these adverse events, both patients completed their participation in the study. Overall, the synbiotic preparation examined in this study seems to be well-tolerated without causing major adverse events. This is in line with observations made for most of the synbiotics (or probiotics) evaluated in clinical IBS trials.

### 4.1. Strengths of the Study

The strength of the presented clinical trial is its design as a randomized, multi-centric, double-blind, and placebo-controlled study performed in a primary-care setting. The study involved IBS patients with a defined stool subtype and disease severity characterized over a relatively long (4 weeks) run-in phase. The treatment duration was 12 weeks, which is at least long enough to avoid the limitation observed in short (2 to 4 weeks treatment) clinical IBS studies. To our knowledge, the present study is the largest IBS study investigating symptomatic improvements in IBS patients treated with a synbiotic preparation. The number of patients included in the study was large enough to determine statistically significant differences between the verum and the placebo groups and allowed even some patient subgroup analyses. Assessment of the effectiveness using the IBS-SSS, IBS-GIS, and IBS-AR scales allows for a comparison of the results of the present study with the data obtained in other IBS clinical trials using these well-defined scales.

### 4.2. Limitations of the Study

The present study used a pragmatic approach, allowing it to be executed with a limited level of involvement of physicians and patients on a fully voluntary basis. This approach obviously comes with a number of limitations. The study included only moderate and severe IBS patients, while no patients with mild forms of IBS were recruited for the study. The patients reported self-assessed symptoms using a weekly patient diary. For the usage of the diary, patients received a short explanation after having been enrolled in the study but were otherwise left alone with this task. Therefore, the data obtained from the patient diary should be interpreted with caution, as there was no ongoing quality control in regard to patient self-reporting of symptoms. No conclusions regarding the lasting effects of the treatment can be made, as follow-up after the end of the 12-week treatment period was not part of the present study design. However, the trial patients are invited for a follow-up visit about 6 months after finishing their participation. Data from these follow-up visits might provide at least some insights into the sustainability of the treatment effects but will have to be interpreted with caution, as they are not part of the registered study design. The impact of the intervention on quality of life was assessed only with the IBS-SSS5 subscale. One of the more complex IBS-QoL-questionnaires (e.g., IBS-QOL, IBS-36) would have been a better choice for assessing the impact of the synbiotic on the quality of life [49,50,51]. However, adding an additional multi-item questionnaire to the study protocol was considered too big of a challenge when designing the trial. Dietary intake is associated with IBS symptoms, so collecting this kind of information would have been interesting. However, to limit the workload for the physicians and patients, no dietary data were collected as part of the present study. Due to budget limitations (there was no external funding for the study), it was not possible to integrate any type of analysis of the fecal microbiota.

## 5. Conclusions

Data from the present study indicate that a complex and balanced mixture of probiotic strains, comprising four *Lactobacilli*, one *Lactococcus*, three *Bifidobacteria*, and a *Streptococcus thermophilus* strain, as contained in the synbiotic tested in this trial, might be an effective and well-tolerated option for treating IBS patients. Future clinical studies with IBS patients will be needed to confirm the efficacy observed in this first trial with this synbiotic preparation. These studies should also include additional measures, e.g., changes in the fecal microbiota, that will help in understanding the underlying mechanism of action resulting in the observed effects. Follow-up assessments of patients who participated in the present study are in the process of being collected. However, a separate randomized clinical study addressing the long-term effects of treatment with the synbiotic will be needed to establish a solid understanding of the sustainability of the treatment effects.

## Figures and Tables

**Table 1:** Composition of the nine-strain synbiotic used in the clinical trial.

Probiotic Strain	Colony-Forming Units (CFU) per Capsule ^1^	% of Total CFU
*Lactobacillus helveticus* SP 27	9.00 × 10^8^	20
*Lacticaseibacillus rhamnosus* Lr-32	4.50 × 10^8^	10
*Lacticaseibacillus casei* Lc-11	2.25 × 10^8^	5
*Lactiplantibacillus plantarum* Lp-115	2.25 × 10^8^	5
*Lactococcus lactis* Ll-23	9.00 × 10^8^	20
*Bifidobacterium longum* Bl-05	6.75 × 10^8^	15
*Bifidobacterium breve* Bb-03	4.50 × 10^8^	10
*Bifidobacterium bifidum* Bb-02	2.25 × 10^8^	5
*Streptococcus thermophilus* St-21	4.50 × 10^8^	10
Total CFU/capsule	45 × 10^8^	100

^1^ Daily dose administered during the treatment was one capsule per day. Each capsule contained 63 mg of fructooligosaccharides (FOS) as a prebiotic component.

**Table 2:** Patient baseline characteristics of placebo and multi-strain synbiotic treatment groups assessed by physicians’ examinations during visit 0 and by analyzing patients’ self-reporting during the run-in phase.

	Placebo (n = 100)	Multi-Strain Synbiotic (n = 101)	*p*-Value
Baseline characteristics assessed by physicians during visit 0			
Weight (kg)	72.1 ± 12.3	70.1 ± 12.3	0.244 ^a^
Height (cm)	172.8 ± 9.5	171.2 ± 9.3	0.252 ^a^
Body-Mass Index (kg/m^2^)	24.0 ± 2.1	23.7 ± 2.1	0.820 ^a^
Age	40.8 ± 10.7	41.9 ± 9.5	0.424 ^a^
Gender (female/male)	55/45	64/37	0.228 ^b^
WGO IBS questionnaire for HCP ^c^	25.0 ± 1.2	24.8 ± 1.6	0.455 ^a^
IBS-SSS ^d^	295.1 ± 23.9	300.5 ± 25.7	0.131 ^a^
IBS severity (moderate/severe) ^e^	57/43	53/48	0.615 ^b^
IBS-SSS1 ^f^	59.1 ± 6.3	59.9 ± 6.2	0.413 ^a^
IBS-SSS2 ^g^	58.3 ± 5.8	58.4 ± 6.0	0.889 ^a^
IBS-SSS3 ^h^	58.7 ± 5.9	60.7 ± 6.1	0.021 ^a^
IBS-SSS4 ^i^	59.3 ± 6.1	59.8 ± 7.8	0.580 ^a^
IBS-SSS5 ^j^	59.8 ± 6.5	61.8 ± 6.1	0.025 ^a^
Baseline characteristics assessed from patients’ reporting during run-in phase			
IBS-Stool Type (D/C/M/U) ^k^	73/20/2/5	73/26/1/1	0.287 ^b^
Bristol Stool Form Scale score IBS-D patients	6.3 ± 0.5	6.4 ± 0.5	0.306 ^a^
Bristol Stool Form Scale score IBS-C patients	1.7 ± 0.5	1.8 ± 0.4	0.386 ^a^

Data are expressed as mean ± standard deviation or numbers. ^a^ Student *t*-test; ^b^ Pearson’s Chi-squared test; ^c^ World Gastroenterology Organisation irritable bowel syndrome questionnaire for health care professionals; ^d^ Irritable Bowel Syndrome Severity of Symptoms Scale; ^e^ moderate IBS: IBS-SSS from 175 to 300, severe IBS: IBS-SSS ˃ 300; ^f^ severity of abdominal pain; ^g^ frequency of abdominal pain; ^h^ severity of flatulence; ^i^ dissatisfaction with bowel habit; ^j^ interference with quality of life; ^k^ IBS-D: IBS with diarrhea, IBS-C: IBS with constipation, IBS-M: IBS with diarrhea and constipation, IBS-U: unspecified IBS.

## Data Availability

The data presented in this study are available upon reasonable request from the corresponding author due to ethical reasons.
